# Construction of Hospital Human Resource Information Management System under the Background of Artificial Intelligence

**DOI:** 10.1155/2022/8377674

**Published:** 2022-08-04

**Authors:** Xiaona Yu, Chunmei Zhang, Chengcheng Wang

**Affiliations:** ^1^Human Resources Department, Qingdao No.8 People's Hospital, Qingdao, 266100 Shandong, China; ^2^Organization and Personnel Section, Qingdao No.6 People's Hospital, 266033 Shandong, China; ^3^Organization and Personnel Section, Qingdao No.9 People's Hospital, 266002 Shandong, China

## Abstract

Under the background of artificial intelligence (AI), a human resource information management system was designed to facilitate hospital human resource management and improve hospital management efficiency. Based on AI, SOA was constructed and Java2 platform enterprise edition (J2EE) was combined with Java to design and research hospital human resource information management system. In addition, the function and performance required by the system were tested. The results showed that the designed system showed high safety in requirement analysis and performance. The function focused mainly on the systematic analysis of personnel management, recruitment management, organization and personnel management, and patient medical information. The constructed system could work normally and achieve the efficiency of hospital human resource management. The evaluation response time of system home page access was less than 1 second when 300 users were concurrent, and the utilization rate of service CPU was lower than 50% without abnormal memory fluctuation. The concurrent response time of all 20 managers online was less than 5 seconds, and the utilization rate of the service was lower than 70%. When the information of 100 employees in the system was queried concurrently, the average CPU utilization of the database server exceeded 90%. After performance optimization, the test result showed that the transaction response time was reduced to 0.23 seconds, which met the target requirement. In conclusion, the proposed intelligent human resource management system could reduce hospital management cost and the high sharing of human resource information provided a reference for the decision-making system of hospital leaders.

## 1. Introduction

A management information system (MIS) is an interpersonal system that integrates the collection, transmission, storage, maintenance, management, processing, output, and use of organization data. MIS can manage and process the organization data and design the processed data into simulation technology and management models by optimization to provide the decisions for hospital decision makers [1, 2]. Human resource management is the foundation and core of hospital management. The demands for hospital resource management by various hospital organizational structures are different. The flexible utilization of human resource management system to realize the requirements of organizational structures with various patterns and to further implement the effective organization and management of data and reduce some unnecessary information sharing is an issue to be addressed currently [3, 4].

Artificial intelligence (AI) is a discipline concerned with the representation of research and the acquisition as well as application of knowledge [5, 6]. AI and each industry involved offer the new impetus for the innovative application of enterprises and industry and the improvement of human life and provide convenience for the acquisition of information [7, 8]. In terms of data analysis, AI can help human resource practitioners become more prospective [9]. Information technology is developed continuously and rapidly; translation medicine, evidence-based medicine, and pharmacoeconomics are rapidly developed; the research of clinical medicine is strongly advocated by the state; and the demand for scientific research still grows constantly [10]. Data informatization can effectively manage the information of nursing staff and plays a certain role in the analysis and decision-making of government departments [11, 12]. Service-oriented architecture (SOA) is a method of designing, developing, deploying, and managing discrete models in computer environment [13–15]. The structure of SOA was simple with high flexibility, and it was widely applied [16]. However, no specialized development language provides technical support for SOA at present. Many scholars combine Java technology with SOA technology to realize information system distribution [17]. Java2 platform enterprise edition (J2EE) of Java could be used to implement Web service to provide better scalability for the system. In addition, it could reduce the coupling between the system and modules more effectively.

In the new stage, the scale of hospitals expands continuously and the number of medical staff increases constantly, which causes some pressures to human resource management. Based on artificial management, traditional methods show low management efficiency, high management costs, and frequent errors. In addition, it is difficult for patients to access information and inconvenient to share information. The utilization of AI technology to actively promote the analysis of medical data and improve its quality and efficiency becomes an urgent problem to be dealt with. Hence, hospital human resource management system was constructed based on AI to the efficiency of hospital human resource management and reduce human resource management cost. Based on the precise and comprehensive development of SOA and functional requirements, a simple and quick interface was provided for users, which provided the reference for the design and development of human resource information management system.

## 2. Data and Methods

### 2.1. Hospital Human Resource Management System

**Figure 1:**
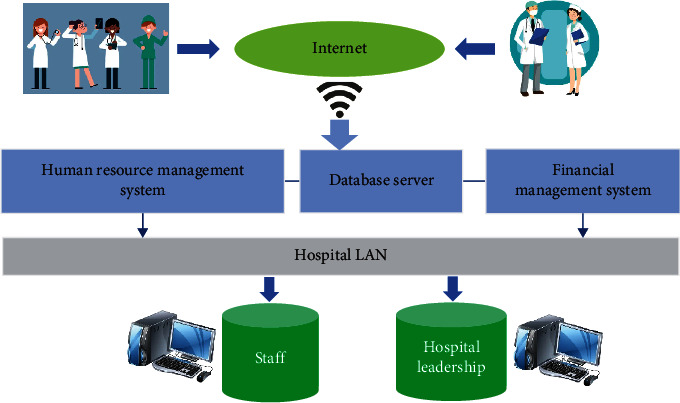
Overall structure of hospital management system.

The hospital human resource management system needs to meet many aspects. General data mainly includes employee transfer management, employee compensation information management, employee basic information management, and employee attendance information management. The design of the management system should not only meet the information sharing of internal personnel but also needs to ensure that extranet management system can access the data in human resource management statistics. The data visualization in the system should better understand and interpret data to enable people to find hidden laws from the seemly chaotic massive data and to provide reference for scientific findings. The overall structure of hospital human resource system is shown in Figure 1 below. The data and resource-related services related to human resource is provided for hospital staff and leaders through hospital local area network (LAN). All hospital employees can access the relevant functions of human resource management system through Internet to accelerate the construction of hospital human resource informatization.

### 2.2. SOA Technology

The main atoms of SOA design include the following contents:

- Explicit interface definition that the interface is stable as well as unambiguous and shows encapsulation
- Self-contained and modularized: the functional entity that implements services is completely independent and autonomous. It can perform deployment, version control, and self-management as well as recovery
- Coarse grain: the number of services should not be excessive. The services rely on message interaction rather than remote procedure call
- Loose coupling: the interdependency and imaging between services, the location of service, implementation techniques, current status, and private data are reduced. The services are invisible to service requests. Interoperability, compatibility, and policy statement are also reduced

**Figure 2:**
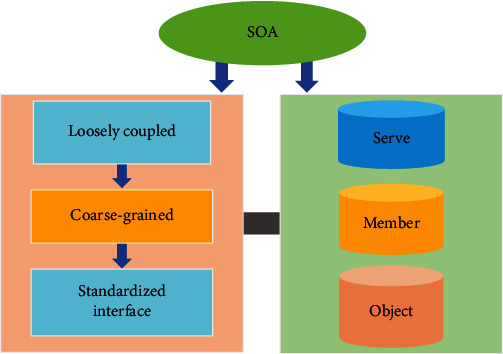
SOA design principles.

Figure 2 illustrated the SOA design principles below.

**Figure 3:**
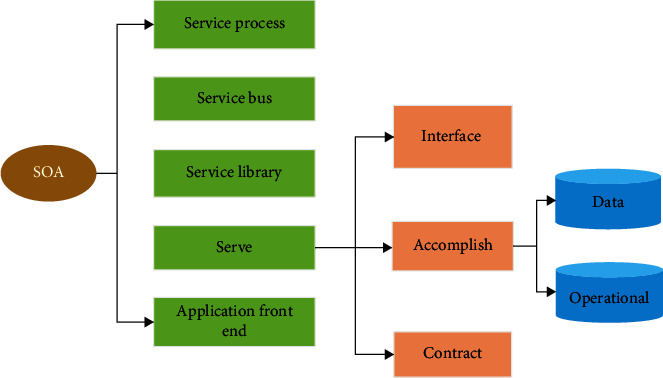
SOA.

SOA is oriented to the integration and development of the model. In a distributed system, the architecture is based on the SOA communication protocol. Each subsystem communicates independently and does not depend on a specific language. The services in the SOA architecture mainly embody the upgrading of functions and realize the concreteness of application front-end functions. The contract carries on the function realization, and the interface is a part of the contract, realizing concrete description of the server realization end to the application degree. The service library is used to realize the execution of independent management and service storage. The requirements of service bus must meet connectivity, technical heterogeneity, communication heterogeneity, and technical service (Figure 3).

### 2.3. Support of SOA by J2EE

**Figure 4:**
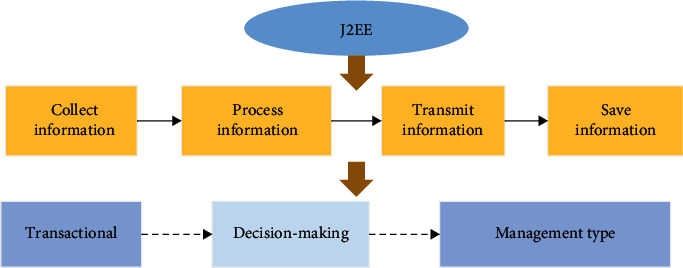
J2EE platform steps and layers.

All major development languages on the market adapt to the support from SOA. Java is featured with “written once, run everywhere,” which meets the requirement of cross-platform operation. The combination of SOA and Java can realize the offline problem of distributed information system. J2EE platform of Java is selected to provide services for Web, which can better achieve the repeatability and extensibility of functions and reduce the coupling between systems and between modules. J2EE focuses on realizing the service support of Web by XML language, which can better achieve SOA and reduce the work intensity of developers. Main J2EE office automation-based steps include information collection, information processing, information transmission, and information saving, as demonstrated in Figure 4 below. The main levels include the transactional level, decision-making level, and management level. Automated office equipment can make use of advanced communication techniques to collect, sort out, process, save, and use information extensively, comprehensively, and rapidly. The advantage of J2EE lies in the use of user interface and enterprise business logic to open with system services in which a series of bottom-level services such as buffer pool and business management are provided. Consequently, developers focus on the business logic of enterprises. The overall complete architecture of J2EE can simplify system solution and consolidate the original advantages of Java standard version. The whole constructed system can significantly reduce the time of delivery to market.

### 2.4. Analysis of Key System Implementation Modules

The construction of performance requirements is a prerequisite of building the system, which requires a clear understanding of performance to improve the quality and pertinence of information management. The construction of the system needs to meet both functional requirements and nonfunctional requirements. The analysis of performance requirements needs to be evaluated from two perspectives, including implementation approach and implementation efficiency. The construction of human resource management system includes the humanization of the main system interface, high processing speed and efficiency of the system, high safety and reliability of the system, the confidentiality and security check mechanisms required by hospital human resource management, the maximum utilization of official free hardware resources and networks, and the reduction of unnecessary system costs. Each page that requires database calls uses this module, and each page in the system becomes more normalized and standard. During the use of the system, link modules need to be called to be connected with data. The specific execution codes of database link modules are as follows.

When the data results queried by users are redundant, the designed system interface should be not only beautiful but also simple. Besides, the queried data should be paged according to certain data, which facilitates user operation. The specific implementation codes are as follows.

The main functions of the current page include data query, page number display, the acquisition of the current page, and skipping to another page.

The query database and the page number of obtaining destination page address are as follows.

During page skipping, corresponding buttons appear on the interface. The page directly skips to the first or last page of the results.

Skipping display codes are as follows:

The experimental environment of the research is carried out in Window7 operating system. The system utilizes Java2 platform and JBuilder8 as the development tool. The language is Mlab7.13 compilation environment. The size of internal storage is 8 G. The main frequency is 3.0 GHz with Intel quadcore.

### 2.5. Performance Test

Performance test was implemented to guarantee the safety, reliability, and execution efficiency of system operation, mainly including link speed test, pressure test, and load test. The connection speed test was performed to test the speed of the connection to application system by users. Too low response speed resulted in the timeout limit on page. Too low connection speed might cause data loss. Besides, users could not get real pages without good experience. Pressure test focused on system limits and the capacity of fault restoration. In other words, the circumstance under which the system crash occurred was tested. If a hacker provided a wrong data load, the system crash occurred. Access right was gained when the system was restarted. Load test was carried out in a reagent network environment. The system allowed a certain number of users to be online at the same time at a certain load level. What would happen when the number of users was exceeded was tested. The system received load test, which ensured that the result was correct and reliable.

The number of parallel connections was set. Under concurrent connections, parallel connection was set. Stress multiplier was the maximum thread, and stress level was the minimum thread. Thread represented the number of threads for request by program system background. In other words, it referred to the simulation of the links of a certain number of clients. According to the loading capacity of the machine, threads were set. In most cases, 200 to 1,000 threads were set. Mock clients were selected within this range. If there were 300 users at a time, the growth of the number of users during the period was analyzed. It was estimated that there were 40 online users, the maximum number of concurrent online users was 10 times, and the maximum number of concurrent online users was 40^∗^10. Besides, the performance or page response index was analyzed.

## 3. Results

### 3.1. Patient Information Page

**Figure 5:**
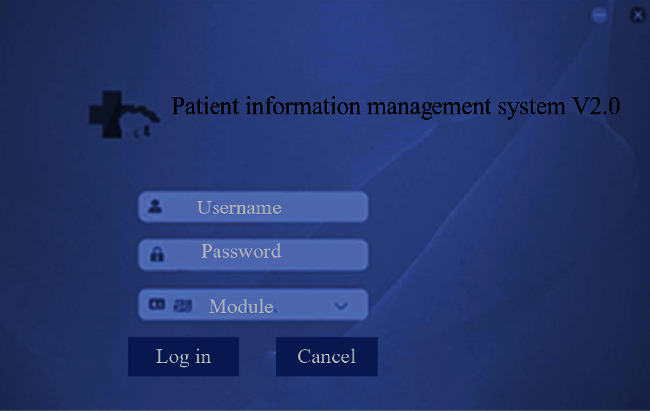
System interface.

In the system, patients can log in the platform to query their personal medical information and learn about their own medical and relevant information of disease diagnosis and treatment anytime and anywhere, which is more conducive to the recovery of diseases.  Figure 5 displayed the login interface of patient personal information management system below.

### 3.2. Login Page

**Figure 6:**
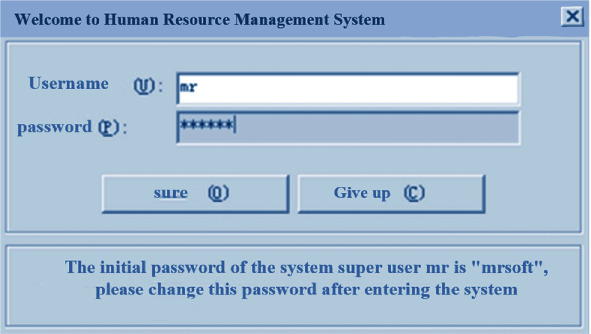
Login interface.

After entering the login page of human resource management system, users can double-click “human resource management system. Exe file icon”. The system login page is displayed in Figure 6 below.

### 3.3. Program Implementation

**Figure 7:**
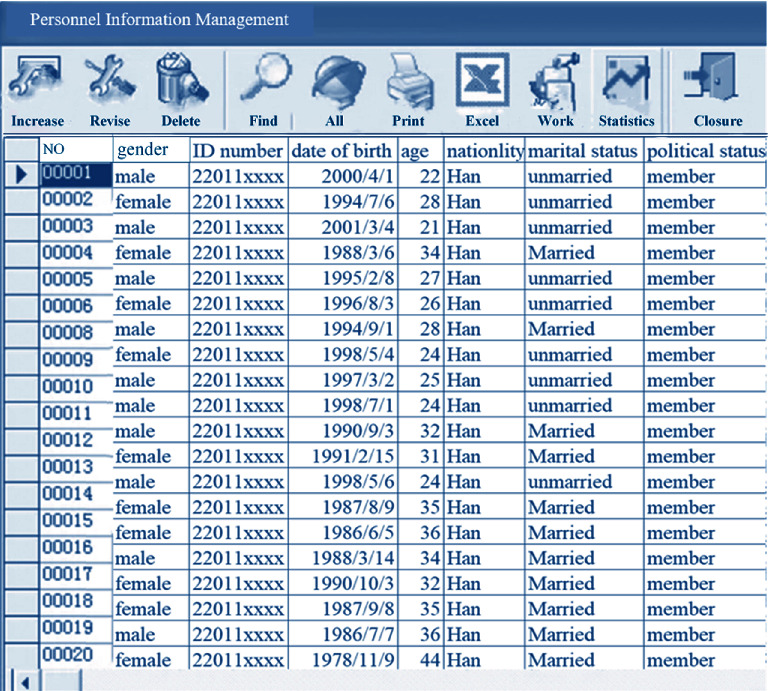
Program implementation interface.

**Figure 8:**
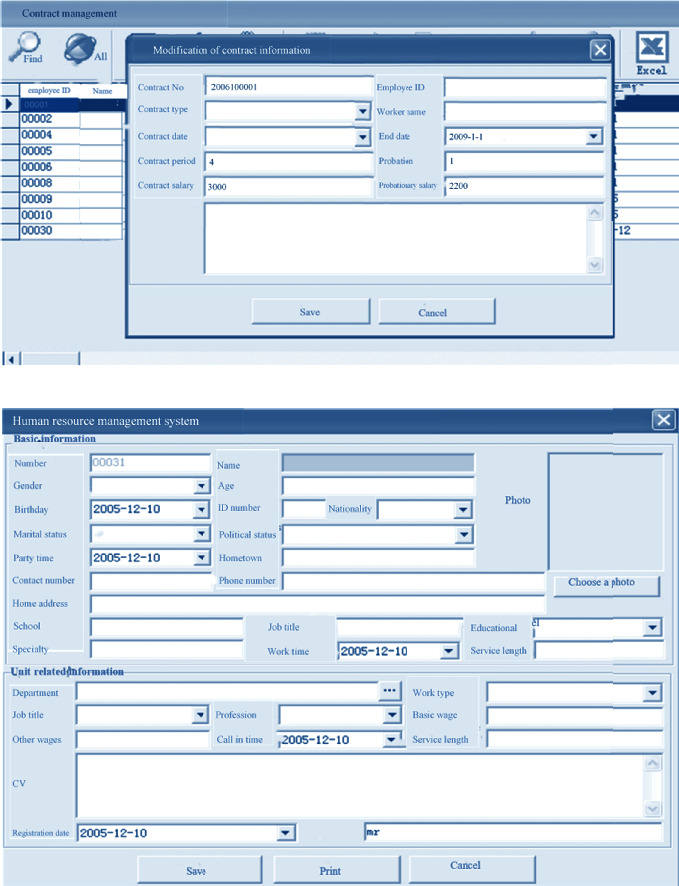
Personnel information adding and contract information modification interface.

After entering the system, the operation on hospital personnel information management items on the main interface, employee transfer, employee turnover, employee reinstatement, training management, and contract management is performed. Different options can be clicked on the interface. Besides, personnel information options can be added, as shown in Figure 7 below.  Figure 8 displayed personnel information adding and contract information modification interface below.

### 3.4. Test Environment

**Figure 9:**
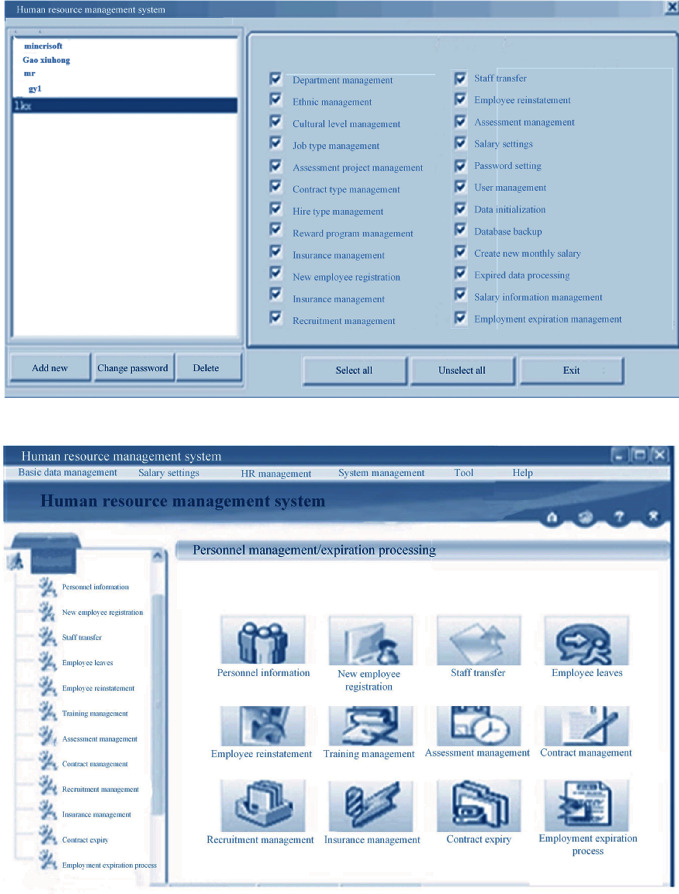
Main interface of human resource management system.

The test is performed from various aspects to check if there are major problems or defects in the software system and to ensure the integrity of the system in fulfilling the requirements of mobile phones. The test cases are the convenient analyzable values input by the system description data of files. During the development of software system, it is necessary to ensure that the system meets user requirements and sufficient detailed design requirements. During the software testing, analog data cover possible participation and are input into the system to obtain the desired output. The correct software system also needs to be broken down and repaired. After the change, the system is retested to ensure the stable operation of the final software system. In Windows XP operating system, the server uses IIS7.0 network, the client end is IE10.0 browser, and the data volume is SQLSERVER 2012. The options in the window of Figure 9 can be used to set permission users. The checkboxes before all functional modules can be selected by selecting “select all” button.

### 3.5. Performance Test Results

The evaluation response time of system home page access was less than 1 second when there were 300 concurrent users. The utilization rate of server CPU was lower than 50% without abnormal memory fluctuation. Besides, the test of function points met the requirements of test targets.

In the system, the response time of 20 concurrent online managerial personnel was less than 5 seconds. The utilization rate of the server was lower than 70%, and the test result also met the requirements of the targets.

When the information of 100 employees in the system was queried concurrently, the average CPU utilization rate of database server exceeded 90% and the average response time was 0.4 s. After performance optimization, the test result demonstrated that the transaction response time was reduced to 0.23 s, which met the requirements of the targets.

## 4. Discussion

AI can work at any time with sufficient energy support. It shows excellent computing power, which enables it to perform thousands of calculations within a few seconds. Computer network mitigation is complex with huge information amount and difficult manual management methods. AI intelligent computing can be utilized to complete information analysis in a short time. Besides, AI can take advantage of advanced programs for information classification as well as automatic and rapid storage and deletion of relevant information. AI can learn and mimic human features and enters a fully restored action with the correct programming [18–20]. The human resource management system is a reasonable allocation of hospital human resources and the scientific management of the automatic calculation of yuan wage, labor insurance, and rewards as well as punishments. The timely and accurate acquisition of personnel-related information and data is realized, and the required reports can be obtained according to different requirements [21, 22]. Client-server application is always adopted as the system architecture of the existing human resource management system. However, the maintenance of the architecture is complex and its tolerance is poor. In addition, the development of the system is difficult with low data security. Many scholars utilized ongoing J2EE to design system architecture for system development and maintenance. Wang et al. [23] adopted the overall architecture Java2 platform J2EE multilayer structure. The interface of the designed human resource management system was simple and comprehensible. The system was operatable with high security. The average response time of business end was shorter than 0.4 seconds. Memory control is below 30%. The designed system was suitable for personnel management. The functionality analysis was performed on the requirements of human resource management to provide the guarantee for the construction and application of hospital human resource management. The constructed system demonstrated high processing speed, security, reliability, and certain fault tolerance. Therefore, it can meet the demands of the hospital human resource management.

The SOA architecture enables integration of existing heterogeneous systems at minimal cost [24]. Based on the above design principles, the overall system was designed using J2EE and established on Java platform. The designed system could not only consolidate the advantages of Java standard edition but also facilitate data storage and retrieval. J2EE offered comprehensive techniques, including xml, ejb, and jsp. All techniques can get good support. By unifying development platforms, J2EE could reduce the costs during the development of multilayer applications. Besides, it could support the existing application programs very well. The performance was enhanced, and security mechanism was implemented. The four-layer model of J2EE consisted of a client-layer component, Web-layer component, business logic layer component, and enterprise information system layer component. In the context of AI intelligence, bottom-level data were automatically accessed. The console of the server could be utilized to implement the deployment of various applications [25]. With the continuous and rapid expansion of hospital scale, it is dispensable to investigate intelligent management mode by combining mainstream informatization. Information intelligentization provides the convenience for the development of information management system. In addition, the successful design of hospital human resource information system is conducive to the effective management of employee and patient information and the improvement of work efficiency of hospitals. The design of information system is never the focus. The design of considerable scholars needs to be updated and upgraded constantly. The human resource management system designed based on SOA in an intelligent way showed high security and good system performance.

## 5. Conclusion

Based on the context of AI, J2EE was combined with Java to carry out the design and research on hospital human resource information management system. In addition, the functions and performances required by the system were analyzed. Based on the analysis of the system, the feasible design was proposed. The results revealed that the constructed system could run normally and could achieve the efficiency of hospital human resource management. The constructed system effectively improved the querying and sharing of human resource information and provided the reference for the decision-making system of hospital leaders. Nonetheless, there were still some limitations in the system. For instance, patients could not implement custom query and print out what they needed. What is more, the fault tolerance of the system should be further strengthened and the processing of some operations was not perfect. The performance of the system in actual problems needed to be further improved.

## Figures and Tables

**Code 1:**
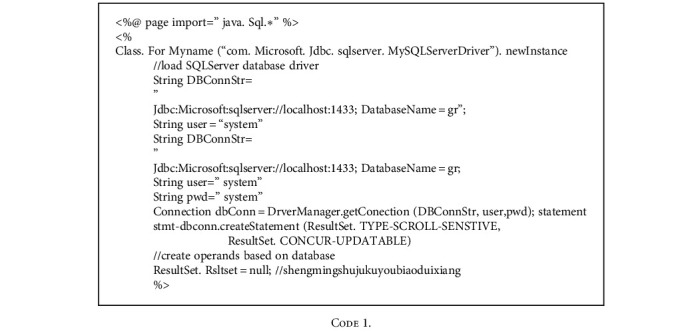


**Code 2:**
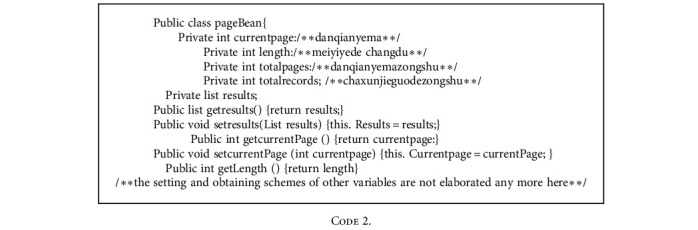


**Code 3:**
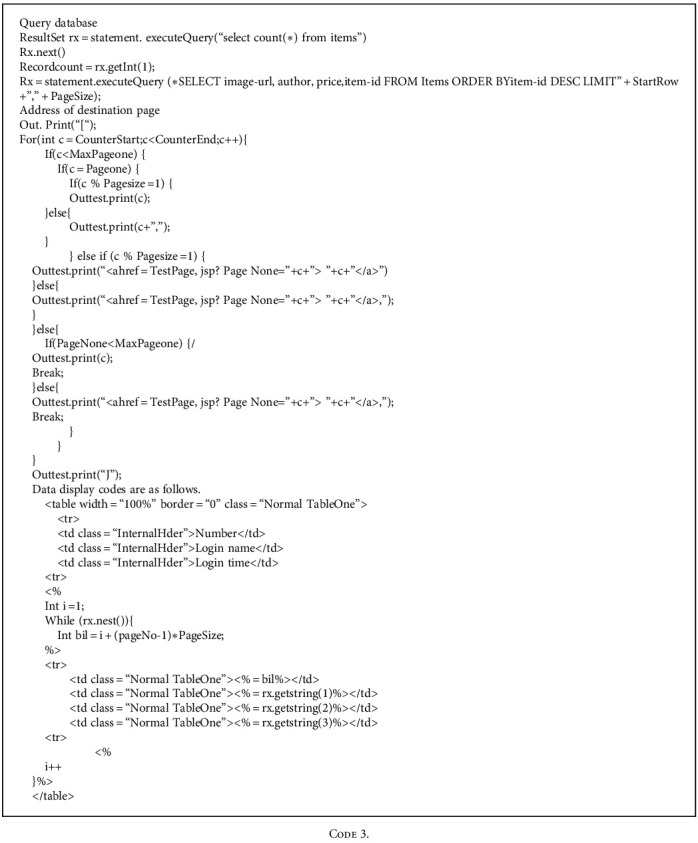


**Code 4:**
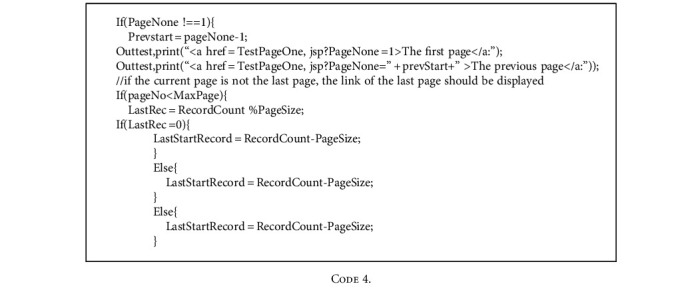


## Data Availability

The data used to support the findings of this study are available from the corresponding author upon request.
